# Pediatric Chronic Kidney Disease During Pandemic Conditions—A Single-Center Experience

**DOI:** 10.3390/jcm14051608

**Published:** 2025-02-27

**Authors:** Łukasz Biesiadecki, Joanna Jacuńska, Paulina Tomecka, Aleksandra Bruciak, Kinga Musiał

**Affiliations:** 1 Students’ Scientific Association, Department of Pediatric Nephrology, Wroclaw Medical University, Borowska 213, 50-556 Wroclaw, Poland; 2 Department of Pediatric Nephrology, Wroclaw Medical University, Borowska 213, 50-556 Wroclaw, Poland

**Keywords:** chronic kidney disease (CKD), congenital anomalies of the kidney and urinary tract (CAKUT), COVID-19, glomerulopathies

## Abstract

**Background/Objectives**: The prevalence of chronic kidney disease (CKD) is increasing worldwide, and this tendency is also visible in pediatric patients. The major clinical challenge is to achieve a diagnosis as early as possible, despite an overt clinical course, especially in the early stages of the disease. Unfavorable external conditions may disturb the proper treatment of chronically ill patients and delay the time of diagnosis. The recent COVID-19 pandemia might have altered the usual diagnostic pathways of different comorbidities, and CKD was probably one of them. However, there are no data on newly diagnosed CKD in children during the time of the pandemia, so our aim was to approach this problem. **Methods**: We analyzed medical records of 154 children with CKD who were hospitalized in the Department of Pediatric Nephrology in prepandemic (years 2015–2019) vs. pandemic and postpandemic (2020–2024) conditions, analyzing the eGFR value and stage of CKD at diagnosis, the underlying diseases leading to CKD, and sex-related differences. **Results**: The number of patients who were diagnosed with CKD in both time periods was comparable. Children hospitalized in the years 2020–2024 presented more often with advanced stages of CKD. The trend towards an increasing share of glomerulopathies, acute kidney injury, and unknown causes of CKD was noticeable under pandemic conditions. **Conclusions**: The COVID-19 pandemic could, probably owing to reduced access to primary healthcare and disrupted routine check-ups, delay the process of diagnosing CKD in children.

## 1. Introduction

The epidemiological assessment of chronic kidney disease (CKD) in children faces two significant challenges. First, there is a paucity of data on the epidemiology of early-stage CKD in the pediatric population, as the condition often progresses asymptomatically, contributing to under-reporting and underdiagnosis in the primary care setting [1]. Second, the clinically silent progression of renal function decline may delay the moment of placing the patient in nephrological care, thus overlooking aggravation to the advanced stages of CKD.

The problem becomes more serious when the numbers are examined. According to data from several European pediatric nephrology societies, the prevalence of early-stage CKD is estimated to be approximately 11–12 cases per million age-related population (pmarp) for stages 3–5 and 8 pmarp for stages 4–5 [2]. A few population-based studies suggest that the prevalence of all-stage CKD in the pediatric population may be as high as 1% [3]. Moreover, the overall incidence of CKD among children has shown an increasing trend for the last 30 years [4].

According to various registries, 40–62% of childhood CKD are due to congenital anomalies of the kidneys and urinary tract (CAKUT) [2,5]. The percentage of candidates whose primary cause of kidney disease is CAKUT has been steadily increasing during a 10-year observation period [6].

The abovementioned trends could have been modified, or even aggravated, by the recent COVID-19 burden, yet there are no publications concerning the impact of pandemic conditions on the CKD epidemiology.

Therefore, our aim was to analyze retrospectively the epidemiology of CKD in children in the last 10 years, taking into account multiple variables at diagnosis: age, kidney function and CKD stage, causative factors of CKD, and sex-related differences, with special focus on the pandemic conditions and their potential influence on CKD in the pediatric population.

## 2. Material and Methods

The medical records of 154 pediatric patients with newly diagnosed chronic kidney disease, hospitalized at the Department of Pediatric Nephrology between 2015 and 2024, were retrospectively analyzed. Patients were stratified into two groups based on the time of diagnosis: prepandemic (2015–2019) and pandemic/postpandemic (2020–2024). Medical records were evaluated for age, gender, CKD stage at diagnosis, and CKD etiology, with comparisons being made between the two timeframes.

The basic demographic data of the studied population are presented in Table 1.

CKD was defined according to the Kidney Disease Outcomes Quality Initiative (KDIGO) guidelines, as either structural or functional kidney damage persisting for at least three months or a reduction in estimated glomerular filtration rate (eGFR) to below 60 mL/min/1.73 m^2^ in children older than two years [5]. CKD was classified into five stages based on the degree of kidney function impairment: stage 1 CKD—eGFR ≥ 90 mL/min/1.73 m^2^; stage 2—eGFR 60–89 mL/min/1.73 m^2^; stage 3—eGFR 30–59 mL/min/1.73 m^2^; stage 4—eGFR 15–29 mL/min/1.73 m^2^; and stage 5—eGFR < 15 mL/min/1.73 m^2^ (Table 2). For children under two years of age, with eGFR values physiologically below 60 mL/min/1.73 m^2^, a CKD diagnosis was made based on the serum creatinine concentration, adjusted to the age-specific reference values. The eGFR values were calculated based on the Schwartz formula [7].

The statistical analysis was performed using Statistica ver. 13 (StatSoft, Tulsa, OK, USA). The chi-square test and Fisher’s exact test were employed to assess the significance of differences between categories. Continuous variables were compared with the use of a nonparametric Mann–Whitney U-test. A *p*-value < 0.05 was considered statistically significant.

## 3. Results

### 3.1. CKD Stage Distribution and eGFR Trends at Diagnosis

**Figure 1:**
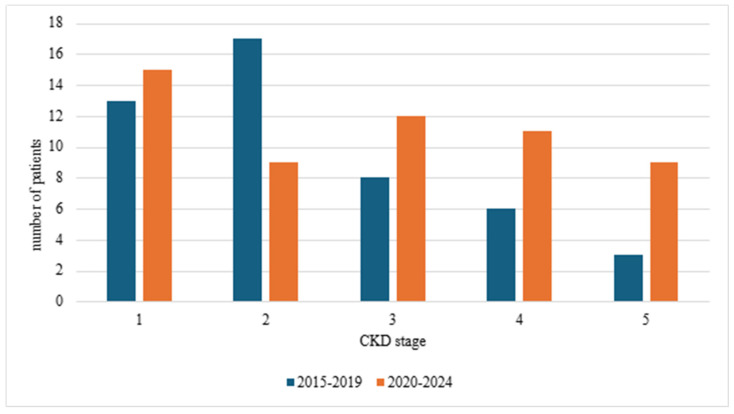
CKD stage at diagnosis in years 2015–2019 and 2020–2024; χ^2^ test *p* = 0.13; CKD—chronic kidney disease.

**Figure 2:**
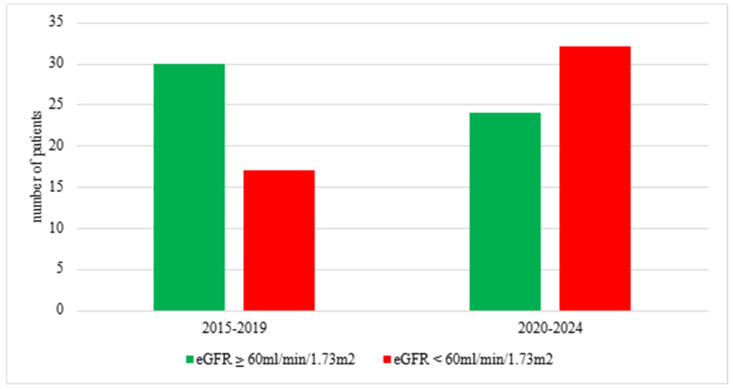
Proportions of patients with mild CKD (eGFR ≥ 60 mL/min/1.73 m^2^) and advanced CKD (eGFR < 60 mL/min/1.73 m^2^) at diagnosis in years 2015–2019 and 2020–2024; χ^2^ test *p* = 0.03; eGFR—estimated glomerular filtration rate; CKD—chronic kidney disease.

The numbers of newly diagnosed CKD patients within the prepandemic (2015–2019) and pandemic/postpandemic (2020–2024) periods were comparable (Table 2). The distribution of CKD stages at diagnosis across these two periods showed no statistically significant difference (Figure 1). However, when stratified according to the eGFR values of < and ≥60 mL/min/1.73 m^2^, there was a statistically significant shift towards the advanced stages of CKD at diagnosis within the 2020–2024 period (Figure 2).

### 3.2. Sex Differences in CKD Population at Diagnosis

**Figure 3:**
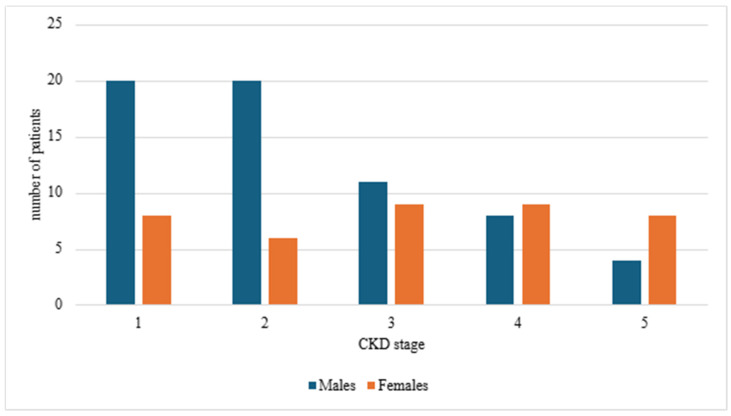
CKD stage at diagnosis in reference to gender in years 2015–2024; χ^2^ test *p* = 0.05; CKD—chronic kidney disease.

There was a trend towards earlier diagnoses of CKD in males (CKD stage 1–2) and a slightly higher proportion of females were diagnosed in the advanced CKD stage (mainly stage 5), but these borderline differences missed statistical significance (Figure 3).

**Figure 4:**
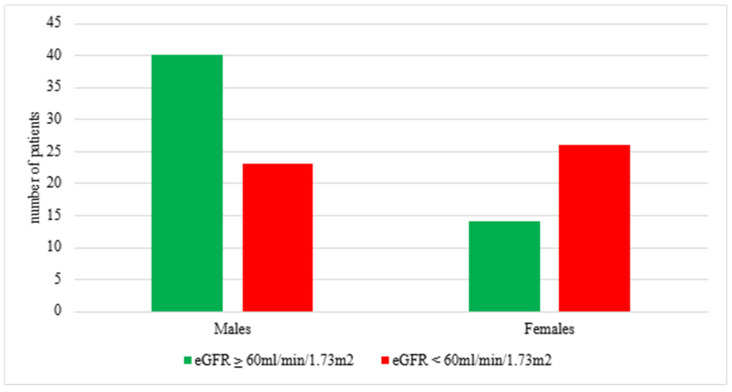
Proportions of males and females with early CKD (eGFR ≥ 60 mL/min/1.73 m^2^) and advanced CKD (eGFR < 60 mL/min/1.73 m^2^) at diagnosis in years 2015–2024; χ^2^ test *p* = 0.004; eGFR—estimated glomerular filtration rate; CKD—chronic kidney disease.

However, the analysis of sex in relation to CKD severity, stratified as mild (eGFR < 60 mL/min/1.73 m^2^) or advanced (eGFR ≥ 60 mL/min/1.73 m^2^), revealed the dominance of males among patients with mild CKD and a higher proportion of females with advanced CKD (Figure 4). This sex-based difference reached statistical significance (*p* = 0.004).

### 3.3. Etiology of CKD in Relation to Age

The proportions of various diseases contributing to the CKD etiology remained similar within the age groups analyzed in the periods of 2015–2019 vs. 2020–2024. In children who were ≤2 years old, CAKUT was the dominant causative factor both before and during/after COVID-19, with a strong prevalence over AKI, genetic, and unknown causes (Table 3). In patients who were >2 years old, CAKUT remained responsible for less than half of CKD cases, whereas AKI, glomerulopathies, and unknown causes increased their shares compared to younger patients, irrespective of the analyzed time period. However, the presence of single-patient representatives in certain categories made the interpretation of these results rather difficult.

**Figure 5:**
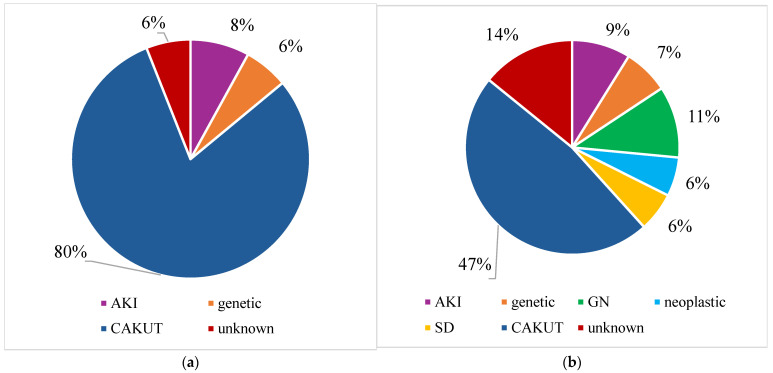
(**a**) CKD etiology in patients aged ≤ 2 years at diagnosis in 2015–2024 period; (**b**) CKD etiology in patients aged > 2 years at diagnosis in 2015–2024 period; CKD—chronic kidney disease; AKI—acute kidney injury; GN—glomerulopathy; SD—systemic disease; CAKUT—congenital anomalies of the kidney and urinary tract.

Therefore, we compared the entire group of children who were diagnosed within the first 2 years of life with those who were diagnosed at an older age in order to examine the age-related discrepancies (Figure 5). In patients who were ≤2 years of age, CAKUT was the most prevalent cause, accounting for 80% of cases. In patients who were >2 years, CAKUT remained the leading cause, but decreased to 48% at the expense of growing incidences of glomerulopathies, systemic diseases, and unknown causes of CKD (Figure 5).

### 3.4. Etiology of CKD in Relation to Sex

CAKUT was the leading etiology of CKD in both analyzed periods and irrespective of sex (Table 4).

**Figure 6:**
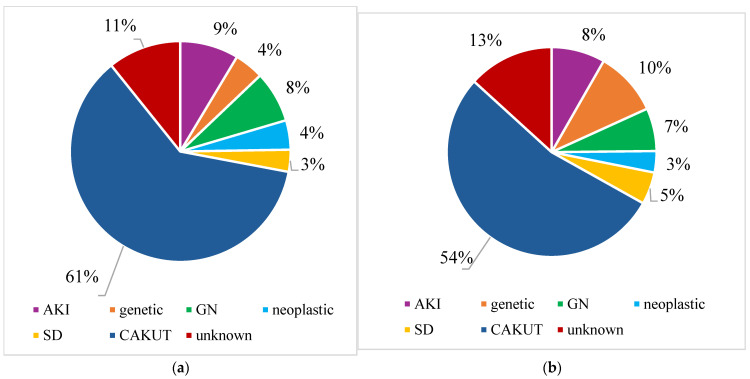
(**a**) CKD etiology in males in 2015–2024 period; (**b**) CKD etiology in females in 2015–2024 period; CKD—chronic kidney disease; AKI—acute kidney injury; GN—glomerulopathy; SD—systemic disease; CAKUT—congenital anomalies of the kidney and urinary tract.

In the years 2015–2019, the higher incidence of CAKUT was related to the male sex of patients, but this trend did not endure during the 2020–2024 period (Table 4). Meanwhile, the proportion of genetic disorders seemed higher in females in both periods (Table 4), but these differences were again difficult to interpret due to the possible bias related to the small numbers of patients (Figure 6).

### 3.5. Etiology of CKD in Relation to the Severity of the Disease

In the pre-COVID-19 era, the number of patients with CKD stage 1–2 at diagnosis exceeded that of children with CKD stage 3–5, whereas in the pandemic and postpandemic periods, the majority of newly diagnosed children with CKD presented with advanced CKD (Table 5).

**Figure 7:**
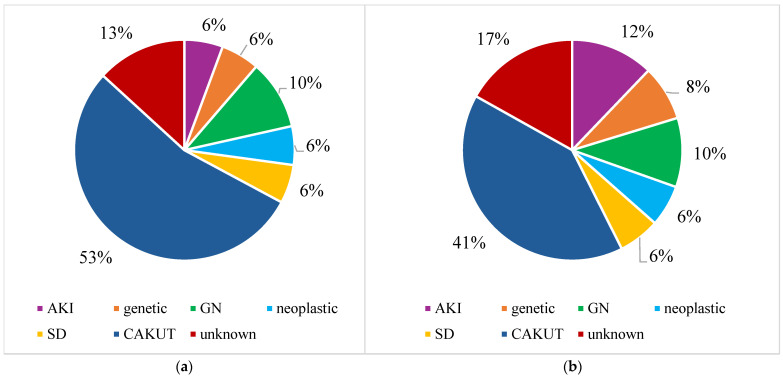
(**a**) Etiology in patients aged > 2 years with early-stage CKD (eGFR ≥ 60 mL/min/1.73 m^2^) at diagnosis in 2015–2024 period; (**b**) Etiology in patients aged > 2 years with advanced CKD (eGFR < 60 mL/min/1.73 m^2^) at diagnosis in 2015–2024 period; CKD—chronic kidney disease; AKI—acute kidney injury; GN—glomerulopathies; SD—systemic diseases; CAKUT—congenital anomalies of the kidney and urinary tract.

The etiology of mild and advanced pediatric CKD at our center has shown a distinct shift between the 2015–2019 and 2020–2024 periods. In mild CKD (eGFR ≥ 60 mL/min/1.73 m^2^), CAKUT remained the most common cause, accounting for nearly half of the cases in the years 2015–2019 and more than a half in the years 2020–2024 (Table 5). A similar increasing trend was found for glomerulopathies (Table 5). When the 2015–2024 period was analyzed as a whole, CAKUT as a main causative factor was followed by cases of unknown origin and glomerulopathies (Figure 7).

In advanced CKD (eGFR < 60 mL/min/1.73 m^2^), the proportion of CAKUT cases decreased in both the pre- and postpandemic periods, mainly at the cost of increasing numbers of unknown etiologies and glomerulopathies (Table 5). The shift from CAKUT towards unknown causes in advanced CKD was also evident when the whole 2015–2024 period was assessed (Figure 7). Moreover, this analysis showed the increasing share of acute kidney injury (AKI) (Figure 7).

### 3.6. COVID-19 as a Causative Factor of CKD

In the analyzed cohort, there were only two patients who developed CKD in the course of COVID-19 as a probable infectious trigger. The first patient was admitted with a suspicion of AKI of an unknown origin and required dialysis. The routine PCR test was negative for COVID-19, but the child had high titers of IgG antibodies. No other infectious agent was identified, and no other probable reason for CKD was revealed. After 3 months, the patient’s renal function improved (dialysis was ceased) but did not normalize, and CKD stage 2 was diagnosed.

Another patient was admitted to our department with a suspicion of AKI, anuria, and high parameters of uremic toxicity. There was no improvement after a few hemodialysis sessions, and symptoms of respiratory tract infection appeared instead. The patient turned out to be COVID-19-positive and was transferred to the ICU because of respiratory insufficiency. After steroid treatment, their general condition improved, but their renal function did not normalize nor improved, and the child remains dependent on dialysis.

## 4. Discussion

The COVID-19 pandemic created unprecedented challenges for healthcare systems worldwide, impacting not only the management of infectious diseases, but also impairing the diagnosis and care of chronic conditions, including CKD. The latter demands early detection, which is essential for slowing down the disease progression and delaying the incidence of complications. Thus, the pandemic may have further aggravated existing diagnostic difficulties, although no data confirming such a tendency exist.

Our data revealed delays in CKD diagnosis among children, confirmed by the significantly higher prevalence of children with CKD with eGFR < 60 mL/min/1.73 m^2^ at diagnosis in the years 2020–2024 compared to the 2015–2019 period. In contrast, the prepandemic conditions provided more chances for early diagnosis of CKD, which was demonstrated by the domination of children with CKD stages 1–2 over those with advanced CKD.

CAKUT remained the major causative factor for CKD, irrespective of the age, sex, or analyzed time period. Its superiority was most evident in the youngest, accounting for 80% of cases in patients under 2 years of age. However, the dominance of CAKUT was still evident in older children. Not surprisingly, this fact translates into the long-term sequelae in the registries of adults with end-stage kidney disease, where CAKUT has become the fourth most common cause of incident kidney replacement therapy [8].

Along with the CKD progression, factors other than CAKUT increased their share under postpandemic conditions. The significant contribution of glomerulopathies was noticeable in mild CKD, whereas an alarming trend of a significant impact of unknown causes and AKI as reasons for CKD dominated when the late stages of CKD were taken into account. All of these could result from late referrals, but recent reviews shed new light on another probable trigger—kidney diseases caused by COVID-19 and able to progress to CKD.

Acute tubular necrosis was reported as the main scenario of renal involvement in the course of SARS-CoV2 infection among adults, while pediatric data confirmed AKI as the most frequent kidney complication of COVID-19 [9,10]. The possible explanation of further progression into CKD could be the concomitance of post-AKI damage and infection-triggered residual inflammation, although these theories have not been confirmed yet [11]. In the case of AKI-related CKD, the lack of regular follow-up in pandemic conditions could result in underdiagnosis or delayed diagnosis of AKI, followed by overlooking the progression into acute kidney disease (AKD) and faster progression to CKD. And yet, the issue is of paramount importance, irrespective of the underlying cause, once it is realized that up to 30% of pediatric AKI aggravates into AKD, which is then followed by CKD development in nearly 10% of cases [12].

A similar argumentation could be raised in the case of the increasing share of glomerulopathies. Collapsing focal segmental glomerulosclerosis was the main post-COVID de novo glomerulopathy among adults, whereas few reports described SARS-CoV2-associated cases of nephrotic syndrome in children [10,13]. No doubt, these data require careful verification and further analysis before an evident cause-and-effect correlation between the increased AKI/GN incidence among children with CKD and COVID-19-related kidney diseases can be proven.

Likewise, there is uncertainty about the probable connection between the increasing share of unknown CKD etiologies and COVID-19’s influence on the faster progression of CKD. The adult data confirmed increased mortality and hospitalization rates among COVID-19 patients with pre-existing CKD, but no proof for faster CKD progression owing to infection exists [14]. Fortunately, the contribution of unknown causes among pediatric CKD patients is still lower than that in the adult population, exceeding 20% [8].

The interesting finding of our study was the dominance of male patients among all children with CKD, irrespective of the time period, age group, or etiological factors. Despite this discrepancy, there was no evidence for a higher incidence of CAKUT among boys with CKD. This finding requires prospective verification in the course of a larger study.

Taken together, these results suggest that pandemic conditions could add to the late referrals of CKD patients but had no major influence on the demographic data. The tendency towards increasing shares of AKI and glomerulopathies in the CKD etiology could be the consequence of COVID-19-related kidney pathologies, but no direct proof for a cause-and-effect relation has been presented so far. No doubt, this trend deserves further follow-up and prospective observations of children with CKD.

While waiting for hard data confirming the abovementioned hypotheses, another probable explanation for late pediatric CKD referrals that is worth considering is whether the healthcare system is deficient under unusual conditions.

According to a nationwide cross-sectional survey in Poland, almost 50% of surveyed adults experienced difficulties in the access to health services, where the main reported barriers included the prolonged waiting time, closure of medical facilities, or their restructuring into dedicated COVID-19 centers [15]. In order to ensure accessibility to healthcare, the Polish government established means of remote medical advice, such as e-visits. Nonetheless, the most affected medical consultations, which decreased in number, included the holiday and night care units in outpatient settings, home visits by physicians, as well as outpatient and home visits by nurses [16]. The public health areas that experienced the largest reduction in services were rehabilitation, screening tests, and periodic health checks, the latter being essential in preventive pediatrics. These limitations may have given way to underdiagnosis of chronic conditions that are characterized by an asymptomatic course until the advanced stages of the disease, such as CKD.

On the other hand, various data revealed a reluctance of self-reporting to healthcare units during the pandemic too. Observations among the healthcare providers in two Manchester hospitals estimated a 24.8% decrease in attendance at the emergency department between February and March 2020 [17]. Other data mentioned that nearly 32% out of 752 pediatricians who were surveyed by the British Paediatric Surveillance Unit and working in April 2020 at pediatric assessment units (PAUs) or emergency departments (EDs) witnessed a patient with a delayed presentation, mostly diabetes mellitus, but also sepsis and malignancy [18]. A further investigation among parents in Ireland documented that nearly 22% refused to attend medical units, although their children required healthcare [19].

No adequate Polish data exist, but pediatric nephrology units continued undisturbed service throughout the whole period of the pandemic and provided consultations for external COVID-19 pediatric patients suffering from nephrological complications. This constant availability of nephrological pediatric service is a probable explanation for the comparable numbers of patients with CKD who were admitted to our department in the 2015–2019 and 2020–2024 periods. And yet, the balance was shifted towards the late stages of CKD, which is pictured by the dramatic discrepancy between the median eGFR values in the years 2015–2019 vs. 2020–2024.

Whereas some publications focus only on the structural burden of the system during the pandemic, the child’s accessibility to proper treatment depends also on the parental factor, resulting in a hesitancy of seeking medical advice under unusual conditions. Those parental concerns in the COVID-19 era could have a multifactorial background, such as misinterpretation of government restrictions of staying at home, fear of being infected during the visit at a medical facility, or an avoidant behavior, exaggerated by the stress of the global pandemic. Thus, the abovementioned reasons could also add to the delayed CKD diagnosis in pediatric patients during the pandemic. Hopefully, lessons taken from this pandemic experience will help avoid future similar obstacles.

On the other hand, the lesson taken from lockdown could be that of limiting COVID-19 spread among children, thus protecting them against SARS-CoV-2-driven CKD. Our two cases of post-COVID-19 CKD have shown severe, or even fulminant, clinical courses with irreversible renal damage shortly after infection.

We have to admit that our study has limitations. This is a single-center experience, so no national conclusions could be made. We did not have full access to the data concerning the availability of general pediatricians during the pandemic, so we can only speculate on the potential impact of restrictions in primary health services on late referrals to specialist care, resulting in a delayed diagnosis of CKD.

And yet, to the best of our knowledge, our study remains the first to investigate the impact of the COVID-19 pandemic on the CKD epidemiology in children. It confirmed the increased incidence of late referrals among children with CKD at the cost of less frequent diagnoses of children with early stages of the disease.

## 5. Conclusions

The process of diagnosing CKD was delayed in children under pandemic conditions. The disruptions caused by the COVID-19 pandemic, including reduced access to routine healthcare, delayed medical evaluations and placed strain on the healthcare system, which may have contributed to this trend. The increased incidence of late referrals could also result from parental behavioral patterns that were triggered by the unusual pandemic conditions. The direct translation of COVID-19-related renal damage into increased incidences of glomerulopathies, AKI, or unknown causes of CKD is yet to be established.

Therefore, further retrospective analyses of healthcare processes during COVID-19, as well as prospective observations of children with CKD who were diagnosed in the pandemic period, are needed to show the full spectrum of connections between different elements of the system and to create a preventive strategy for prospective crisis situations.

## Figures and Tables

**Table 1:** Basic demographic data of patients with chronic kidney disease (CKD).

	2015–2019	2020–2024
**Number of all CKD patients**	74	79
Males %	59	62
Females %	41	38
**Number of patients ≤ 2 years old**	27	23
Males %	63	57
Females %	37	43
**Number of patients > 2 years old**	47	56
Males %	57	64
Females %	43	36

**Table 2:** Proportions of patients with CKD stages 1–5 at diagnosis in years 2015–2019 and 2020–2024.

	2015–2019	2020–2024
Number of patients > 2 years old	47	56
Median eGFR [ml/min/1.73 m^2^] (IQR)	73 (50–91)	42 (22–99) #
Children with CKD stage 1 [%]	28	26
Children with CKD stage 2 [%]	36	16
Children with CKD stage 3 [%]	17	21
Children with CKD stage 4 [%]	13	20
Children with CKD stage 5 [%]	6	16

CKD—chronic kidney disease; eGFR—estimated glomerular filtration rate; IQR—interquartile range; # *p* < 0.05 Mann–Whitney U test.

**Table 3:** Etiology of chronic kidney disease (CKD) in pre- and pandemic/postpandemic conditions according to the patients’ age.

	2015–2019	2020–2024
**Patients ≤ 2 years old [N]**	27	23
CAKUT [N/%]	20/75	19/84
AKI [N/%]	2/7	2/8
Genetic [N/%]	3/11	1/4
Unknown [N/%]	2/7	1/4
**Patients > 2 years old [N]**	47	56
CAKUT [N/%]	22/47	27/48
AKI [N/%]	4/9	5/9
Genetic [N/%]	3/6	4/7
GN [N/%]	3/6	8/14.5
Neoplastic [N/%]	3/6	3/5
SD [N/%]	5/11	1/2
Unknown [N/%]	7/15	8/14.5

CAKUT—congenital anomalies of the kidney and urinary tract; AKI—acute kidney injury; GN—glomerulopathy; SD—systemic disease.

**Table 4:** Etiology of chronic kidney disease (CKD) in pre- and pandemic/postpandemic conditions according to the patients’ gender.

	2015–2019	2020–2024
**Females [N]**	30	30
CAKUT [N/%]	15/50	17/57
AKI [N/%]	2/7	3/10
Genetic [N/%]	2/7	2/7
GN [N/%]	4/13	2/7
Neoplastic [N/%]	1/3	1/3
SD [N/%]	3/10	0
Unknown [N/%]	3/10	5/16
**Males [N]**	44	49
CAKUT [N/%]	28/64	29/59
AKI [N/%]	4/9	4/8
Genetic [N/%]	½	3/6
GN [N/%]	½	6/13
Neoplastic [N/%]	2/5	2/4
SD [N/%]	2/5	½
Unknown [N/%]	6/13	4/8

CAKUT—congenital anomalies of the kidney and urinary tract; AKI—acute kidney injury; GN—glomerulopathy; SD—systemic disease.

**Table 5:** Etiology of chronic kidney disease (CKD) in the years 2015–2019 and 2020–2024 in relation to the severity of CKD.

	2015–2019	2020–2024
**Mild CKD (eGFR ≥ 60 mL/min/1.73 m^2^) [N]**	30	24
CAKUT [N/%]	14/46	14/59
AKI [N/%]	3/10	2/8
Genetic [N/%]	2/7	¼
GN [N/%]	2/7	4/17
Neoplastic [N/%]	3/10	2/8
SD [N/%]	2/7	¼
Unknown [N/%]	4/13	0
**Advanced CKD (eGFR < 60 mL/min/1.73 m^2^) [N]**	17	32
CAKUT [N/%]	8/46	13/41
AKI [N/%]	1/6	3/9
Genetic [N/%]	1/6	3/9
GN [N/%]	1/6	4/13
Neoplastic [N/%]	0	1/3
SD [N/%]	3/18	0
Unknown [N/%]	3/18	8/25

CAKUT—congenital anomalies of the kidney and urinary tract; AKI—acute kidney injury; GN—glomerulopathy; SD—systemic disease.

## Data Availability

The generated datasets are available from the corresponding author on reasonable request.

## References

[1] Plumb L., Sinha M.D., Jones T., Redaniel M.T., Ridd M.J., Owen-Smith A., Caskey F.J., Ben-Shlomo Y. (2025). Identifying children who develop severe chronic kidney disease using primary care records. PLoS ONE.

[2] Harambat J., van Stralen K.J., Kim J.J., Tizard E.J. (2012). Epidemiology of chronic kidney disease in children. Pediatr. Nephrol..

[3] Harambat J., Madden I., Hogan J. (2021). Épidémiologie de la maladie rénale chronique chez l’enfant [Epidemiology of pediatric chronic kidney disease]. Nephrol. Ther..

[4] Zhao W.-M., Li X.-L., Shi R., Zhu Y., Wang Z.-J., Wang X.-R., Pan H.-F., Wang D.-G. (2024). Global, regional and national burden of CKD in children and adolescents from 1990 to 2019. Nephrol. Dial. Transplant..

[5] Kidney Disease: Improving Global Outcomes (KDIGO) CKD Work Group (2024). KDIGO 2024 Clinical Practice Guideline for the Evaluation and Management of Chronic Kidney Disease. Kidney Int..

[6] Lentine K.L., Smith J.M., Miller J.M., Bradbrook K., Larkin L., Weiss S., Handarova D.K., Temple K., Israni A.K., Snyder J.J. (2023). OPTN/SRTR 2021 Annual Data Report: Kidney. Am. J. Transplant..

[7] Schwartz G.J., Munoz A., Schneider M.F., Mak R.H., Kaskel F., Warady B.A., Furth S.L. (2009). New equations to estimate GFR in children with CKD. J. Am. Soc. Nephrol..

[8] Ortiz A., Kramer A., Ariceta G., Rodriguez Arevalo O.L., Gjerstad A.C., Santiuste C., Trujillo-Aleman S., Ferraro P.M., Methven S., Santamaría R. (2024). Inherited kidney disease and CAKUT are common causes of kidney failure requiring kidney replacement therapy: An ERA Registry study. Nephrol. Dial. Transplant..

[9] Muthya A., Ekinci E.I., Lecamwasam A. (2024). What is the spectrum of kidney pathology associated with COVID-19?. Intern. Med. J..

[10] Baek H.S., Cho M.H. (2023). Kidney complications associated with COVID-19 infection and vaccination in children and adolescents: A brief review. Clin. Exp. Pediatr..

[11] Nlandu Y.M., Tannor E.K., Bamikefa A.T., Makulo J.-R.R. (2024). Kidney damage associated with COVID-19: From the acute to the chronic phase. Ren. Fail..

[12] Chisavu F., Gafencu N., Stroescu R., Chisavu L., Schiller A. (2024). Outcomes of acute kidney injury continuum in children. J. Nephrol..

[13] Qi L., Deep A., Fox J., Yii M., Rahman M., Myint M., Myat H., Thet Z. (2025). A scoping review on adult patients with de novo glomerular diseases following COVID-19 infection or vaccine. Int. Urol. Nephrol..

[14] Lin Y.-C., Lai T.-S., Lin S.-L., Chen Y.-M., Chu T.-S., Tu Y.-K. (2021). Outcomes of coronavirus 2019 infection in patients with chronic kidney disease: A systematic review and meta-analysis. Ther. Adv. Chronic Dis..

[15] Mularczyk-Tomczewska P., Zarnowski A., Gujski M., Jankowski M., Bojar I., Wdowiak A., Krakowiak J. (2022). Barriers to accessing health services during the COVID-19 pandemic in Poland: A nationwide cross-sectional survey among 109,928 adults in Poland. Front. Public Health.

[16] Mrożek-Gąsiorowska M., Tambor M. (2024). How COVID-19 has changed the utilization of different health care services in Poland. BMC Health Serv. Res..

[17] Isba R., Edge R., Jenner R., Broughton E., Francis N., Butler J. (2020). Where have all the children gone? Decreases in paediatric emergency department attendances at the start of the COVID-19 pandemic of 2020. Arch. Dis. Child..

[18] Lynn R.M., Avis J.L., Lenton S., Amin-Chowdhury Z., Ladhani S.N. (2021). Delayed access to care and late presentations in children during the COVID-19 pandemic: A snapshot survey of 4075 paediatricians in the UK and Ireland. Arch. Dis. Child..

[19] Nicholson E., McDonnell T., Conlon C., Barrett M., Cummins F., Hensey C., McAuliffe E. (2020). Parental Hesitancy and Concerns around Accessing Paediatric Unscheduled Healthcare during COVID-19: A Cross-Sectional Survey. Int. J. Environ. Res. Public Health.

